# Apigenin Inhibits the Histamine-Induced Proliferation of Ovarian Cancer Cells by Downregulating ERα/ERβ Expression

**DOI:** 10.3389/fonc.2021.682917

**Published:** 2021-09-08

**Authors:** Manman Liu, Yani Zhang, Qiqi Xu, Guirong Liu, Na Sun, Huilian Che, Tao He

**Affiliations:** ^1^Key Laboratory of Precision Nutrition and Food Quality, Key Laboratory of Functional Dairy, Ministry of Education, College of Food Science and Nutritional Engineering, China Agricultural University, Beijing, China; ^2^National Engineering Research Center of Seafood, School of Food Science and Technology, Dalian Polytechnic University, Dalian, China; ^3^Beijing Key Laboratory of Plant Protein and Cereal Processing, College of Food Science and Nutritional Engineering, China Agricultural University, Beijing, China; ^4^Zhongguancun International Medical Inspection and Certification Co. Ltd, Beijing, China

**Keywords:** ovarian cancer, mast cells, histamine, estrogen receptor, apigenin

## Abstract

**Background:**

Apigenin (APG), a natural flavonoid, can affect the development of a variety of tumors, but its role in ovarian cancer remains unclear. There has been an increasing amount of evidence supporting the vital role played by mast cells and the bioactive mediators they release, as components of the tumor microenvironment, in the progression of ovarian cancer (OC); however, the mechanism warrants further exploration.

**Methods and Results:**

In this study, a combination of transcriptomics analysis and application of TCGA database was performed, and we found that the expression of genes related to mast cell degranulation in ovarian cancer tissues changed remarkably. We then explored whether histamine, a major constituent of mast cell degranulation, could affect the development of ovarian cancer through immunohistochemistry analysis and cell proliferation assays. The results showed that a certain concentration of histamine promoted the proliferation of ovarian cancer cells by upregulating the expression of estrogen receptor α (ERα)/estrogen receptor β (ERβ). Additionally, we found that the inhibition of ERα or the activation of ERβ could inhibit the proliferation of ovarian cancer cells induced by histamine through real-time PCR and western blot assays. Finally, we demonstrated the attenuation effect imparted by apigenin in histamine-mediated ovarian cancer *via* the PI3K/AKT/mTOR signaling pathway.

**Conclusion:**

Our research revealed that apigenin decelerated ovarian cancer development by downregulating ER-mediated PI3K/AKT/mTOR expression, thus providing evidence of its applicability as a potentially effective therapeutic agent for ovarian cancer treatment.

## Introduction

As one of the most commonly reported cancers among women, ovarian cancer (OC) ranks first in mortality among gynecological tumors, and is characterized by a high recurrence, thus establishing itself as a grave risk to the lives and health of women worldwide (1). There seems to be a multifactorial contribution to this disease, such as psychology, environment, the expression of oncogenes, and the inhibition of tumor suppressor genes; however, its pathogenesis remains unknown (2). Currently, there are many clinical treatment approaches available for ovarian cancer. Among them, the most widely used approach is tumor cytoreductive surgery combined with platinum therapy or paclitaxel administration (3). Although surgery and chemotherapy using platinum-based treatments promote better survival in patients with advanced or metastatic ovarian cancer, most patients continue to present with deterioration of their case; therefore, there is an urgent need to explore and develop new therapeutic targets for this debilitating disease.

As components of the tumor microenvironment, mast cells (MCs) play a complex role in promoting angiogenesis, tissue remodeling, and in regulating the host immune response by enabling the secretion of proteases to trigger the activation of receptors, prostaglandins, histamine (HA), and other mediators, thereby promoting tumor growth (4). Among them, histamine, a low-molecular-weight biogenic amine, is the main bioactive substance released upon the degranulation of MCs. Its expression and synthesis have been detected in various cancer cells and malignant tumors (5). The action of histamine is realized after its binding to different receptors (6); the binding induces different responses pertaining to cell proliferation through different signaling pathways. Studies have shown that the histamine content of breast tumor tissue is positively correlated with the number of MCs, which indicates that MCs are the source of histamine in tumor tissues. The increased activity of L-histidine decarboxylase, an enzyme that catalyzes the production of histamine, was observed in certain tumors, including colon cancer, ovarian cancer, endometrial cancer, and melanoma, *via* immunohistochemistry and RT-PCR, and a high level of histamine release was observed *via* radioimmunoassays (7–9). Similarly, a considerable number of mast cells were observed in colorectal cancer tissues, and cancer cells near histamine-producing cells were observed to possess a high proliferation ability, and such aspects were found to negatively impact the overall prognosis of patients (10). Therefore, the impact of histamine on the development and prognosis of ovarian cancer should be further explored.

Apigenin (APG) is a natural plant flavonoid compound that is widely present in celery, chamomile, olive, thyme, and other plants (11). In the 1960s, apigenin first attracted the attention of the scientific community because of its ability to inhibit the release of histamine from basophils (12). Studies have shown that in addition to its antioxidant and anti-inflammatory effects (13), APG can inhibit cell proliferation in pancreatic, colorectal, lung, and other tumors, characterized by a reduction in the expression of Hypoxia Inducible Factor-1 (HIF-1), Vascular Endothelial Growth Factor (VEGF), and their receptors in the tumor environment (14).

In this study, we conducted a bioinformatics analysis based on RNA-Seq and TCGA database and *in vitro* studies to investigate the role of histamine and apigenin in OC, and found that APG attenuated the excessive proliferation of HA-induced OC cell lines. Our results showed that APG mitigated the HA-induced proliferation of OC cells by regulating the ER-mediated PI3K/AKT/mTOR pathway, which might provide insights into the development of potential treatment strategies for ovarian cancer.

## Materials and Methods

### Ovarian Tissue Specimens

The ovarian cancer tissues used in this study were collected from hospitals in Beijing that extended cooperation with our research team. The experiment complied with the relevant regulations and was approved by Human Research Ethics Committee. Six fresh tissues were analyzed using RNA-Seq technology; the tissues were derived from patients who underwent radical ovarian cancer surgery in the hospital from January 2015 to December 2016, including those derived from three cases of healthy ovary tissue and three cases of serous ovarian tumors. The information of patients are given in the **Supplementary Table 1**. All tissues were stored at -80°C before being subjected to further analyses.

### RNA Extraction and Transcriptome Sequencing

Total RNA extraction from frozen tissues was conducted using the TRNzol reagent (TransGen Biotech, Beijing, China). After the performance of quality control, at least 3 µg RNA with RNA integrity number (RIN) greater than 8.0 was used for database construction. A 100-bp paired-end RNA sequencing experiment was performed using the HiSeq2500^®^ system (Illumina, USA). The database construction and sequencing services were provided by Novogene (Beijing, China).

### OC and Normal Control Datasets

Data retrieved from TCGA were used for the integrated analysis in this study. RNA-Seq data derived from patients with OC (n=308) in TCGA and normal samples (n=88) in the GTEx database were obtained from UCSC Xena (https://xenabrowser.net/datapages/). Raw data were downloaded and normalized using a robust multi-array average (RAM) method using the “affy” package in R software.

### Differential Analysis and Functional Enrichment Analysis

The RNA sequencing reads were aligned to the reference sequence information provided by the Ensembl project using TopHat based on the Bowtie algorithm (15). Using the limma package, the differentially expressed genes were determined, and the genes with *P* < 0.05, and |log_2_(fold change)| > 1 were selected as the significantly differentially expressed genes (DEGs). To better understand the function of the selected DEGs, we used the clusterprofiler package of R to perform GO function enrichment analysis and KEGG pathway enrichment analysis. Statistical significance was set at *P*<0.05.

### Cell Culture and Treatments

Human ovarian cancer cell lines OVCAR-3 and Anglne were provided by the BeNa Culture Collection and Procell Life Science & Technology Co., Ltd, respectively, which were sourced from the American Type Culture Collection. The OVCAR-3 and Anglne cells were routinely cultured in dulbecco’s modified eagle medium (DMEM) containing 10% FBS and 1% penicillin/streptomycin/amphotericin B and were maintained in a humidified incubator at 37°C with 5% CO_2_. The cells were subjected to treatment with different concentrations of histamine (1, 5, 10, 50, and 100 ng/mL) and apigenin (0.1, 0.5, 1, 5, and 10 μM) for 48 h. Additionally, 1 μM AZD9496, PHTPP, PPT, and DPN were added to the treatment group and incubation was observed for 48 h.

### Immunohistochemistry

Tissue specimens were embedded in paraffin and cut into 4 μm-thick sections. After performing high-temperature retrieval at 98°C with antigen, tumor sections were incubated with the respective antibodies and biotin-conjugated secondary anti-mouse IgGs. The tissues were stained with diaminobenzidine and visualized using the electron microscopy protocol proposed by Soslow (16).

### Cell Proliferation Assay

Cell proliferation was measured using the CCK-8 kit (Beyotime, Beijing, China). Briefly, 2×10^4^ OVCAR-3 and Anglne cells suspended in 100 μL culture medium were inoculated into 96-well culture plates and cultured in a humidified incubator at 37°C with 5% CO_2_ for 12 h to enable cell adherence to the plate. Then, different doses of histamine (100, 50, 10, 5, and 1 ng/mL) were added to the treatment wells. After culturing under the same conditions for 48 h,10 μL of the CCK-8 reagent was added. After incubation for 1 h, the absorbance was measured at 450 nm using a microplate reader. The cell proliferation rate was calculated as follows: 

$$\text{Proliferation rate }(\%)=\frac{\text{OD sample}-\text{OD blank}}{\text{OD control}-\text{OD blank}}\times 100$$

### mRNA Expression Analysis *via* Real-Time PCR

Total mRNA isolation from OVCAR-3 cells was performed using Trizol Up reagent (TransGen Biotech) and samples were reverse-transcribed using a cDNA reverse transcription kit (Tiangen Biotech) according to the manufacturer’s instructions. Quantitative real-time PCR (qRT-PCR) analyses of histamine receptors and ERs were performed using the TransStart^®^ Green qPCR SuperMix (Bio-Rad Laboratories, California, USA). The primer pairs used for the amplification of the target genes are presented in **Supplementary Table 2**.

### Protein Abundance Analysis *via* Western Blotting

Briefly, the cells subjected to treatments were homogenized in lysis buffer and centrifuged at 14,000 × *g* for 20 min to collect the supernatants. Protein concentrations were quantified using the Bradford protein assay kit (Beyotime). Cell proteins were separated on a 12% polyacrylamide gel and transferred onto polyvinylidene fluoride membranes. Then, the membranes were blocked with freshly prepared 5% skimmed milk powder in TBS/Tween-20 buffer for 2 h, following which they were washed and incubated overnight at 4°C with the specific primary antibodies. Thereafter, horseradish peroxidase-conjugated secondary antibodies were incubated with the membranes for 2 h. Proteins were visualized using chemiluminescence reagents, and the signals were detected using a gel documentation system.

### Statistical Analysis

All data in the experiment were analyzed using the SPSS 13.0 and GraphPad Prism 5 software. The experimental data are expressed as mean ± SD from three independent biological replicates. Changes observed in the experiment were assessed for statistical significance using one-way analysis of variance (ANOVA). Values were considered significant at *P*<0.05.

## Results

### Mast Cell Degranulation May Expedite the Progression of Ovarian Cancer

**Figure 1:**
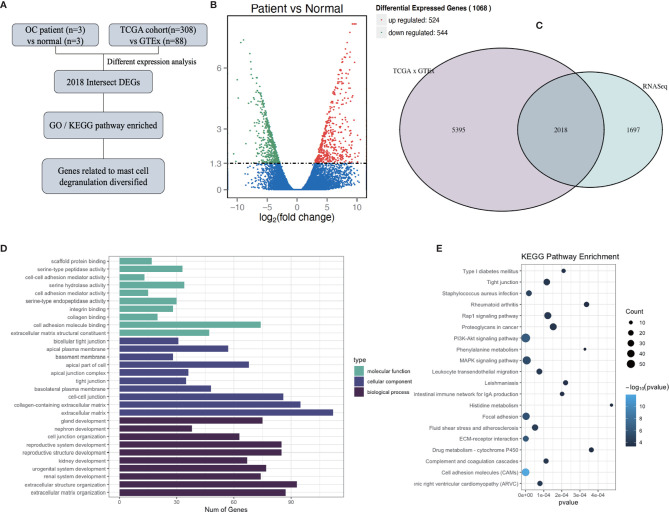
Determination of genes differently expressed between normal ovarian tissue and ovarian cancer tissue. **(A)** Flow diagram of current bioinformatics research: a combination of RNA-Seq analysis and TCGA database. **(B)** Volcano plot of all genes differentially expressed between normal ovarian tissue (n = 3) and ovarian cancer tissue (n = 3), at a significance thresholds of |log2(Fold Change)| > 1, *P*adj<0.05. **(C)** Venn diagram of the results of the analyses of RNA-Seq and differently expressed genes (DEGs) in TCGA and GTEx. **(D, E)** GO **(D)** and KEGG **(E)** enrichment pathway analysis involving intersection DEGs.

To investigate the influence of mast cell degranulation on ovarian cancer, the differentially expressed genes between normal ovary tissues and ovarian cancer tissue were analyzed according to the count values of all detected genes (**Figure 1A**). After quality evaluation, the sequencing results were observed to satisfy the requirements, as shown in **Supplementary Figure 1** and **Supplementary Table 3**. A total of 1,068 differentially expressed genes (DEGs) were identified at a cutoff of |log_2_(fold change)| > 1, *P*adj<0.05, and included 524 upregulated genes and 544 downregulated genes (**Figure 1B**). To increase the credibility of the results, we downloaded OC transcriptome data from TCGA database and selected 2,018 genes with a significance level of *P*_ _<0.05, and |log_2_(fold change)| > 1 at intersection with our sequencing results (**Figure 1C**). **Supplementary Table 4** depicts the top 20 differentially expressed intersection genes. To further analyze the biological functions and signaling pathways of the 2,018 DEGs, GO and KEGG enrichment analyses were performed. GO analysis mainly concentrated on the extracellular matrix, collagen-containing extracellular matrix, extracellular structure organization, extracellular matrix organization, and cell-cell junction, among others. KEGG pathway analysis indicated the presence of PI3K-Akt signaling pathway, MAPK signaling pathway, human papillomavirus infection, among others (**Figures 1D, E**).

After the intersection DEGs were screened and analyzed, the DEGs associated with mast cells were filtered and were observed to play a role in the growth, migration, and degranulation of mast cells, as listed in **Table 1**. The increased expression of vesicle-associated membrane protein-8 (VAMP-8), a fusion protein of the soluble N-ethyl maleimide sensitive factor (NSF) adhesion protein receptor (SNARE) family, garnered our attention, which regulates exocytosis (17). Meanwhile, as a member of the glysin superfamily, the protein CapG is reportedly involved in the remodeling of muscle fibers and in the regulation of endocytosis by binding with Ca^2+^ and by adding caps to the F-actin extension terminal (18). These findings suggest that ovarian cancer may be related to the degranulation of MCs.

**Table 1:** Differentially Expressed Genes Associated with Mast Cell.

GENETIC ID	Log2 Fold Change	p-VALUE	GENE SYMBLE
ENSG00000254087	1.816986385	2.19E-48	LYN
ENSG00000165025	3.166230266	1.95E-111	SYK
ENSG00000213658	-1.249033065	6.30E-37	LAT
ENSG00000173402	1.360901575	9.86E-37	DAG1
ENSG00000167323	-1.88400827	1.15E-62	STIM1
ENSG00000003137	-1.366819395	9.11E-38	CYP26B1
ENSG00000042493	3.258830431	1.18E-120	CAPG
ENSG00000104783	1.904277241	2.11E-35	KCNN4
ENSG00000118640	4.104054766	9.88E-192	VAMP8
ENSG00000128342	1.136883578	2.37E-18	LIF
ENSG00000113594	-1.935188331	1.13E-77	LIFR
ENSG00000125538	1.750439678	9.64E-38	IL1B
ENSG00000135373	4.265317351	7.55E-121	EHF
ENSG00000117020	-3.119062492	1.84E-107	AKT3
ENSG00000169554	-3.19147177	1.28E-133	ZEB2
ENSG00000134853	-5.128618444	2.54E-160	PDGFRA
ENSG00000107562	-1.55706417	2.22E-16	CXCL12
ENSG00000005249	-2.317857566	1.91E-78	PRKAR2B
ENSG00000185291	-1.103999101	1.10E-65	IL3RA
ENSG00000100385	1.509685457	4.46E-31	IL2RB

### The Key Facilitating Role of Histamine in the Proliferation of Ovarian Cancer Cells

**Figure 2:**
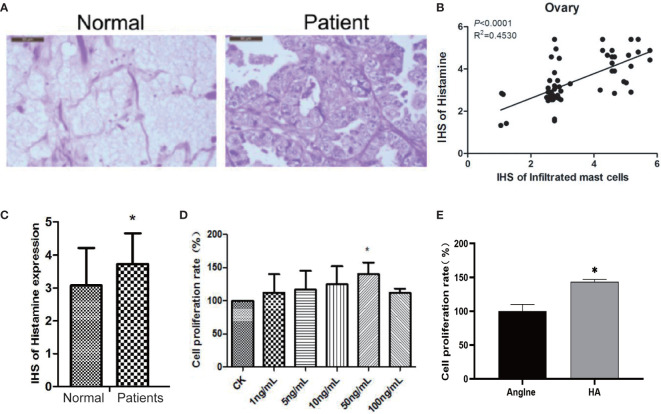
Histamine released by mast cell degranulation stimulates the proliferation of ovarian cancer cells. **(A)** HE staining of normal ovarian tissue (left) and ovarian cancer tissue (right). **(B)** Correlation between mast cell infiltration and histamine release in ovarian cancer tissue. **(C)** Histamine release levels in normal ovarian and ovarian cancer tissues, *P < 0.05 (compared to normal tissues). **(D)** OVCAR-3 cell proliferation after treatment with different concentrations of histamine. **(E)** Anglne cell proliferation rate after treatment with histamine at a concentration of 50 ng/mL. *P < 0.05 (compared to untreated Anglne cell group).

It is not clear whether histamine, the main active constituent released during the degranulation of MCs, plays a role in the development of ovarian cancer. Therefore, to determine the relationship between histamine release and the development of ovarian cancer, we analyzed histamine levels in normal ovary tissues and ovarian cancer tissues *via* immunohistochemical staining. The results indicate that compared with normal ovary tissue, tumors in ovarian cancer tissues are poorly differentiated, with more cell nuclei and conspicuous interstitial infiltration. Histamine levels in cancer tissues were significantly higher than those in normal tissues (*P<0.05) (**Figures 2A–C**). These results suggest that high levels of histamine release in tumor tissues may be related to the formation or development of tumors.

Next, we used the human ovarian cancer cell line OVCAR-3 for further experiments. OVCAR-3 cells were subjected to treatment with 100, 50, 10, 5, and 1 ng/mL histamine for 48 h, with no treatment considered as a control, the proliferation rate of which was recorded as 100%. As shown in **Figure 2D**, the proliferation of OVCAR-3 ovarian cancer cells was significantly increased when subjected to co-culture with 50 ng/mL histamine (*P < 0.05), similar to the phenomenon observed in Anglne cells (**Figure 2E**). Taken together, these results indicate that histamine at a certain concentration accelerates the proliferation of tumor cells.

### Histamine Promotes the Proliferation of Ovarian Cancer Cells by Regulating Estrogen Receptor Expression

**Figure 3:**
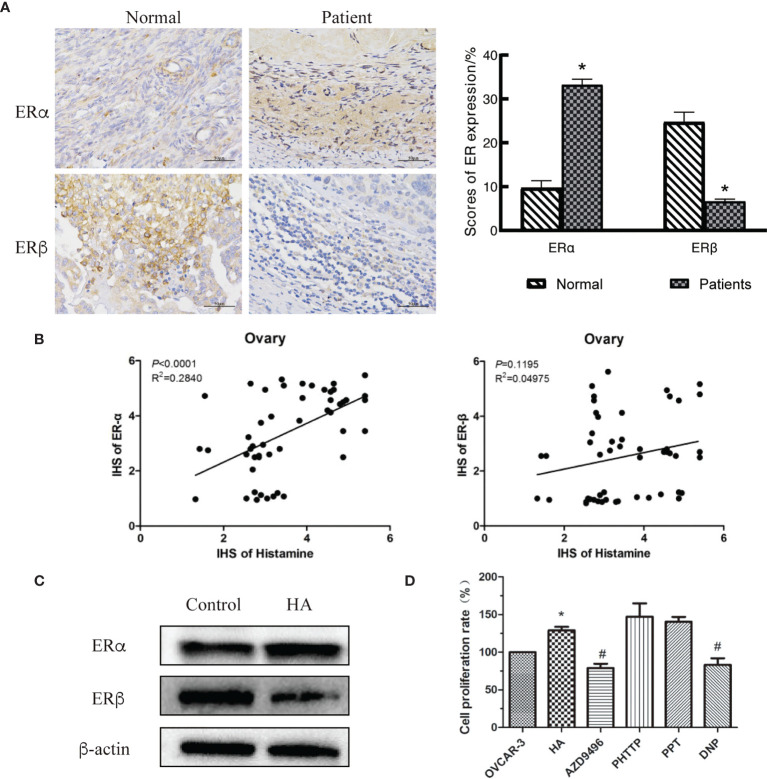
Histamine induces the development of ovarian cancer by disordering expression of estrogen receptors (ERs). **(A)** Immunohistochemical detection of ovarian tissues, *P < 0.05 (compared to normal tissues). **(B)** Correlation analysis between histamine and ERα (left)、ERβ (right) in ovarian cancer tissues. **(C)** Western blot analysis of the expression of ERα and ERβ in histamine-treated OVCAR-3 cells. **(D)** Effect of Inhibiting or activating of estrogen receptors on the proliferation of OVCAR-3 cells. AZD9496, PHTTP, PPT, DNP are respectively ERα inhibitors, ERβ inhibitors, ERα agonists, ERβ agonists; *P <0.05 (Compared to untreated OVCAR-3 cell group); ^#^ *P* < 0.05 (compared with the HA-treated group).

Activation of estrogen-related signaling pathways is an important condition for the development of gynecological tumors since estrogen performs biological functions by binding to its receptors (18). There are two subtypes of estrogen receptors (ERs), namely ERα and ERβ. Different ERs play distinct roles in various tumor types, stages, and development phases (19–21). Bioinformatics analysis showed that the estrogen receptor gene was differentially expressed in ovarian cancer tissues (**Table 2**). Therefore, we speculated that HA contributed to the development of ovarian cancer tumors by regulating the expression of ERs. We measured the expression levels of ERα and ERβ in normal ovary and ovarian cancer tissues and observed that the expression level of ERα in ovarian cancer tissues was significantly increased, while the level of ERβ was decreased (**Figure 3A**).

**Table 2:** The Expression of ERs Relative Genes.

GENETIC ID	Log2 Fold Change	p-VALUE	GENE SYMBLE
ENSG00000091831	1.03703487	1.59E-13	ESR1
ENSG00000140009	-2.208519987	1.52E-173	ESR2

We also investigated the relationship between histamine, ERα, and ERβ in human tissues. In ovarian tissues, HA was significantly and positively correlated with the expression of ERα (*P*<0.0001), but was not correlated with ERβ expression (*P*=0.1195) (**Figure 3B**). The poor correlation between HA and ERβ may be limited by the small number of samples in this study. Western blotting was performed to examine the expression of ERs in HA-treated cells. Results showed that the expression of ERα was upregulated, while the expression of ERβ was downregulated in HA-treated cells (**Figure 3C**). To further verify whether histamine played a tumor-promoting effect through estrogen receptors, AZD9496 (an ERα inhibitor), PHPPT (an ERβ inhibitor), PPT (an ERα agonist), and DNP (an ERβ agonist) were used to subject OVCAR-3 cells to the desired treatment (**Figure 3D**). As depicted, treatment with AZD9496 and DNP, but not PHPPT or PPT, significantly reversed the histamine-induced increase in ovarian cancer cell proliferation (#*P*<0.05). This finding supports and adds credibility to the hypothesis that histamine may accelerate the development of ovarian cancer through the differential expression of ERα and ERβ.

### Apigenin Inhibits the Histamine-Induced Proliferation of Ovarian Cancer Cells by Regulating the Expression of Estrogen Receptors

**Figure 4:**
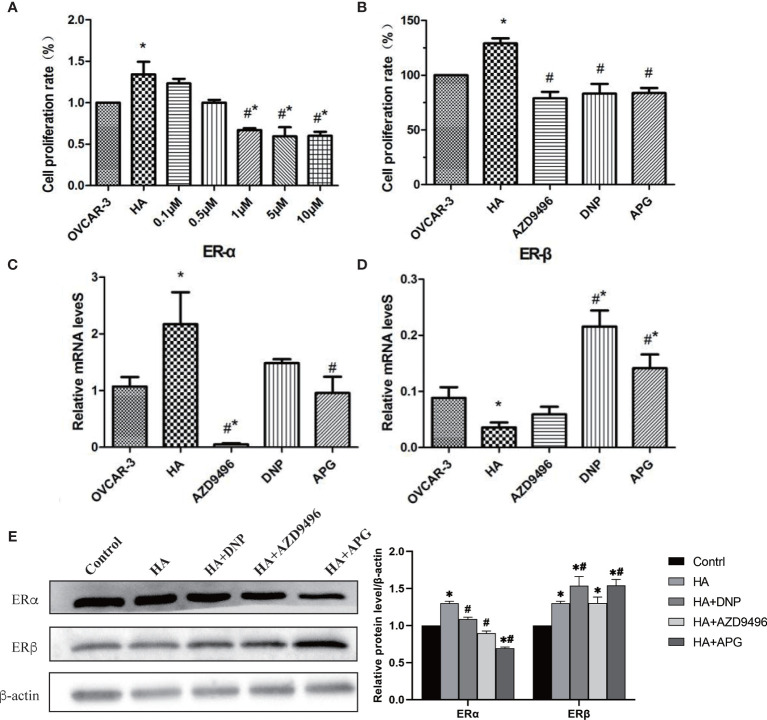
Apigenin inhibits the tumor-promoting effect of histamine by down-regulating ERα/ERβ. **(A, B)** The effect of different concentrations of apigenin, AZD9496, and DNP on the proliferation of OVCAR-3 cells. The concentration of AZD9496 and DNP was 1 µM. **(C, D)** The mRNA expression of ERα **(C)** and ERβ **(D)**. **(E)** The protein expression of ERα and ERβ by western blot. *P < 0.05 (Compared to untreated OVCAR-3 cell group); #*P* < 0.05 (compared with the HA-treated group).

Previous studies have shown that apigenin inhibits the development of a variety of gynecological tumors (22, 23); however, whether it can inhibit ovarian cancer induced by histamine remains unclear. Therefore, we first investigated whether apigenin inhibited the proliferation of OVCAR-3 cells after histamine treatment. Apigenin at concentrations of 0.1, 0.5, 1, 5, and 10 μM was added to the co-culture of histamine (50 ng/mL) and ovarian cancer cells. The results revealed that OVCAR-3 cell proliferation was inhibited by the introduction of apigenin at different concentrations in a dose-dependent manner. Apigenin at concentrations of 1, 5, and 10 μM remarkably suppressed the proliferation of ovarian cancer cells compared with the histamine-treated group (#*P*<0.05) (**Figure 4A**, **Supplementary Figure 2**), which proved that after histamine treatment, apigenin inhibited the proliferation of tumor cells. Additionally, 1 μM APG was selected for addition for subsequent experiments, which was the lowest concentration where apigenin demonstrated remarkable efficacy.

Previous studies have shown that histamine promotes the proliferation of ovarian cancer cells by regulating the expression of ERs, and this can be inhibited by using the ERα inhibitor AZD9496 and the ERβ agonist DNP. As a natural flavonoid, apigenin affects the proliferation of ovarian cancer cells by targeting the estrogen receptor. We then compared the antitumor activity of apigenin by conducting treatment of ovarian cancer cells with AZD9496 and DNP. Our results indicated that apigenin effectively inhibited the histamine-induced proliferation of OVCAR-3 cells, similar to the effect shown by AZD9496 and DNP treatment (**Figure 4B**).

We also determined whether apigenin suppressed histamine-induced tumor cell proliferation by affecting the expression of the histamine H1 (HRH1) or H3 receptor (HRH3). Results showed that the mRNA levels of HRH1 and HRH3 were not significantly changed by apigenin treatment, indicating that apigenin did not inhibit the histamine-induced increase in tumor cell proliferation by affecting the expression of HRH1 and HRH3 (**Supplementary Figure 3**). We then investigated whether apigenin affected the mRNA and protein expression levels of ERα and ERβ in OVCAR-3 cells (**Figures 4C–E**). The results showed that after HA treatment, the expression level of ERα increased significantly and that of ERβ decreased (*P*<0.05). However, apigenin treatment reversed this change and resulted in the establishment of almost normal levels, both at the transcriptional and translational levels.

### Apigenin Inhibits the Histamine-Induced Proliferation of Ovarian Cancer Cells *via* the PI3K/AKT/mTOR Pathway

**Figure 5:**
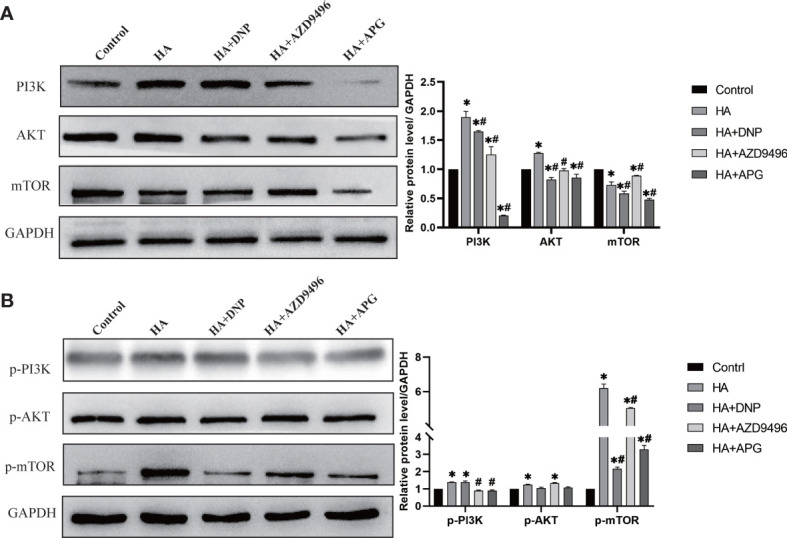
Apigenin inhibits ovarian cancer induced by histamine through attenuating PI3K/AKT/mTOR pathway. **(A)** Western blot analysis of PI3K, Akt and mTOR expression level of differently-treated OVCAR-3 cells. **(B)** Western blot analysis of p-PI3K, p-Akt and p-mTOR expression level. *P < 0.05 (Compared to untreated OVCAR-3 cell group); ^#^ *P* < 0.05 (compared with the HA-treated group).

Studies have indicated that ERs can establish interactions with PI3K to regulate cancer development (24). In our study, the enrichment of the PI3K/AKT/mTOR signaling pathway played a considerable role in the progression of ovarian cancer (**Figure 1E**). Therefore, we investigated whether the change in the expression of ERs induced by apigenin could suppress the PI3K/AKT/mTOR pathway. As shown in **Figure 5**, the overexpression of PI3K, AKT, and mTOR caused by histamine was attenuated after treatment with apigenin. Additionally, the activation of p-PI3K, p-AKT, and p-mTOR was also inhibited, similar to the effect observed with the treatment involving the use of ER modulators DPN and AZD9496. These findings demonstrated that the suppressive effects of apigenin on the proliferation of ovarian cancer were mediated by the PI3K/AKT/mTOR pathway.

## Discussion

Mast cells are granular cells of the innate immune system that play a role in orchestrating adaptive immune responses. They are well known for their role in allergic reactions following IgE-mediated activation of the high-affinity cell surface-expressed IgE receptor FcεRI (25). MCs participate in many physiological and pathological reactions of the body, mainly through the secretion and release of active mediators and cytokines, also known as degranulation (26). As mast cell degranulation plays a key role in mediating the immune-inflammatory response, it has been used as a target for the treatment of IBD, IBS, and other diseases. Carroll found that the scores of abdominal pain-related behaviors in stress-sensitive Wistar Kyoto rats were decreased after treatment with disodium cromoglycate (DSCG), suggesting that stabilization of the MCs and limitation of their degranulation contributed towards amelioration of visceral hypersensitivity (27). Studies have revealed that MCs also function in the tumor microenvironment (28). Therefore, we screened the differentially expressed genes of MCs between normal ovary tissue and ovarian cancer tissue to assess the potential impact of MC degranulation on the development of ovarian cancer. Spleen tyrosine kinase (Syk) plays an essential role in IgE receptor signaling (FcεRI), which leads to mast cell degranulation. Divalent binding of the tandem SH2 domain (tSH2) of Syk to the intracellular ITAM motif of FcεRI activates the kinase domain of Syk, thereby initiating cell degranulation (29). As a member of the Src family of non-receptor protein tyrosine kinases, Lyn is the first kinase that is activated after FcεRI cross-linking, and its high expression indicates mast cell degranulation (30). Consistent with findings reported in previous studies, variations in the retinoic acid degrading enzyme Cyp26b1 (31), macrophage capping protein CapG (32), calcium-activated potassium channel protein KCNN4 (33), VAMP-8 (17), and leukemia inhibitory factor (LIF) (34) confirmed the accuracy of the RNA-seq results, suggesting that MC degranulation might expedite the progression of ovarian cancer.

Histamine, a low-molecular-weight biogenic amine, is the most important substance released upon mast cell degranulation. Histamine exhibits a strong vasodilative effect and increases the permeability of capillary and venule walls, leading to the development of local tissue edema (27). Massive mast cells were observed in colorectal cancer tissues, and cancer cells near histamine-rich regions were found to be highly proliferative, leading to a poorer prognosis (35). Next, we explored whether the tumor-promoting effect mediated by mast cell degranulation was derived from histamine. Immunohistochemical analysis of histamine in normal human and cancerous tissues and the proliferation of OVCAR-3 cells determined the effect of histamine on tumor development. Presently, there have been many reports on the effects of histamine on tumors. Notably, most malignant cell lines and experimental tumors highly express L-histidine decarboxylase (HDC), a type of histamine synthase, and contain high concentrations of endogenous histamine, which can collectively regulate a variety of biological responses related to tumor growth through paracrine or autocrine signaling into the extracellular medium (36), as well as angiogenesis, cell invasion, migration, differentiation, apoptosis, death, and the immune response, indicating that histamine may be considered a key mediator in the development and progression of cancer. Many *in vivo* studies have reported the use of animal models with homologous or xenogeneic melanoma grafts to prove that both endogenous and exogenous histamine can stimulate tumor growth (37), an observation which is consistent with our findings.

As a hormone-dependent tumor, the development of ovarian cancer is closely related to the levels of estrogen and its receptor. It is generally believed that ERβ is tumor-suppressive, whereas ERα is reportedly tumor-inductive. Our previous studies have found that histamine induces cervical cancer tumor growth by regulating the expression of ERs (38). Therefore, we examined the expression of Erα and ERβ in human ovary tissues and determined the correlation between ERs and OC. The proliferation of ovarian cancer cells is accelerated in the presence of ERα but is inhibited when ERβ levels increase. This was consistent with the findings reported by Chan who stated that ERβ was abnormally expressed in various estrogen-dependent tumors (such as breast cancer and prostate cancer) and resulted in a reduction in cell movement and invasion, leading to increased tumor cell apoptosis (39). Therefore, we report that the different relative expression levels of ER subtypes in individual tumors may produce different effects.

**Figure 6:**
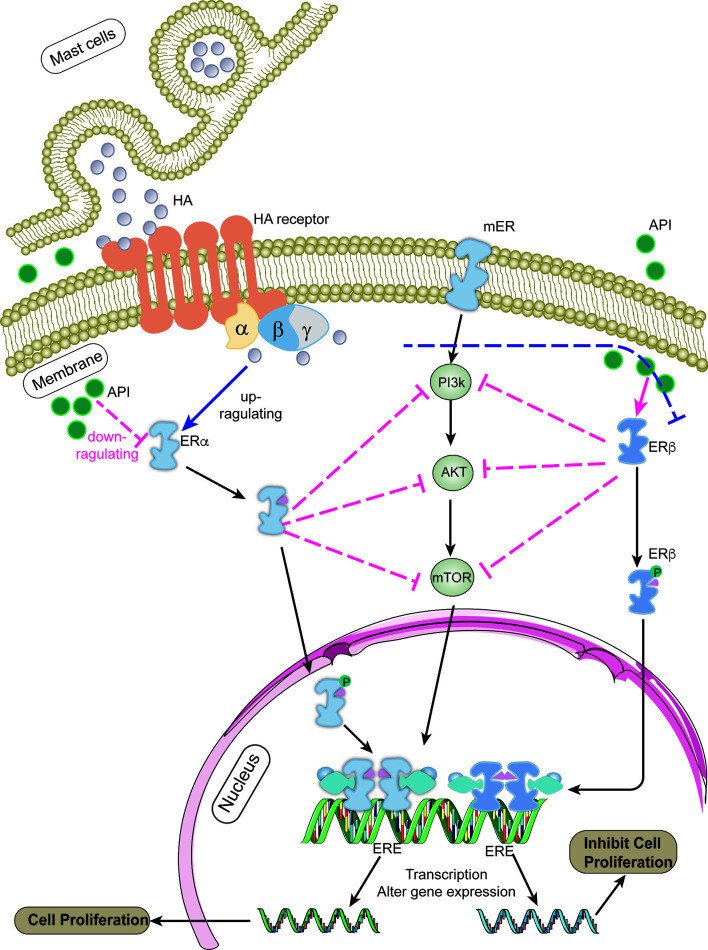
The mechanism of apigenin for inhibiting the proliferation of ovarian cancer cells. After histamine binds to its receptors, it up-regulates the expression of ERα and down-regulates the expression ERβ (blue line). ERs binds to the estrogen response element (ERE) sequence of the target gene promoter after dimerization (38), activating the transcription of downstream target gene (40, 41), such as cell cycle regulators (CCND1, c-Myc) and apoptosis-related proteins (BCL2, BCLXL), etc., which works on promoting cell proliferation and tumor development. Apigenin suppresses PI3K/AKT/mTOR signaling pathway by regulating the expression level of ERα/ERβ (red line), thereby attenuates the development of ovarian cancer induced by histamine.

Apigenin, a natural flavonoid, can decelerate the growth of many types of tumors; however, its anti-tumor mechanism remains unclear. Our results confirmed that apigenin could inhibit the proliferation of OVCAR-3 tumor cells induced by histamine, similar to that observed after AZD9496 and DNP treatment. The results of the mRNA expression analysis indicated that apigenin exerted no effect on the expression of histamine receptors, but exhibited a certain regulatory effect on the expression of ERs, the mechanism of which has been illustrated in **Figure 6**. It has been proven that apigenin possesses estrogen-like properties. Apigenin treatment can increase the number of endometrial glands, stimulate cell growth, and increase the weight of the uterus after establishing interactions with estrogen receptors (42). Apigenin exerts its anti-tumor effects through a variety of mechanisms, including cell growth inhibition, apoptosis induction, cell cycle inhibition, angiogenesis inhibition, cancer proliferation inhibition, and regulation of the expression of oncogenic proteins (43). The PI3K pathway is also closely related to the transcription and expression of ERs. Studies have shown that inhibition of the PI3K pathway in ER-positive breast cancer results in increased ER-dependent transcriptional activity (44).

In summary, apigenin affects the PI3K/AKT/mTOR signaling pathway by regulating the expression level of ERα/ERβ, thereby decelerating tumor development promoted by histamine. Our study shows that apigenin may be deemed a potential natural compound that can be used to inhibit ovarian cancer progression; however, its specific mechanism should be further explored.

## Data Availability Statement

The data presented in the study are deposited in the NCBI Sequence Read Archive, accession number PRJNA757121.

## Ethics Statement

The studies involving human participants were reviewed and approved by China Agricultural University Human Research Ethics Committee. The patients/participants provided their written informed consent to participate in this study. Written informed consent was obtained from the individual(s) for the publication of any potentially identifiable images or data included in this article.

## Author Contributions

Conceptualization, QX and YZ. Data curation, ML, QX and YZ. Formal Analysis, ML, GL, QX. and YZ; Methodology, QX, GL and ML. Funding acquisition, HC. Supervision, HC, TH and NS. Writing – original draft, ML, QX and YZ. Writing – review & editing, ML, HC and TH. All authors contributed to the article and approved the submitted version.

## Funding

This work was supported by Chinese Nutrition Society-Zhongshi Yingke Nutrition Research Fund (CNS-FF2019A21).

## Conflict of Interest

Author TH was employed by company Zhongguancun International Medical Inspection and Certification Co. Ltd.

The remaining authors declare that the research was conducted in the absence of any commercial or financial relationships that could be construed as a potential conflict of interest.

## Publisher’s Note

All claims expressed in this article are solely those of the authors and do not necessarily represent those of their affiliated organizations, or those of the publisher, the editors and the reviewers. Any product that may be evaluated in this article, or claim that may be made by its manufacturer, is not guaranteed or endorsed by the publisher.

## Supplementary Material

Supplementary Figure 1 Determination of DEGs between normal ovarian and ovarian cancer tissues though RNA-Seq analysis. **(A)** Correlation coefficient diagram between normal ovarian (n = 3) and ovarian cancer tissue samples (n = 3). **(B)** Principal Component Analysis diagram between ovarian tissue samples; **(C, D)** GO **(C)** and KEGG **(D)** enrichment analysis of DEGs.

Supplementary Figure 2 The proliferation rate of Anglne cell after HA and APG treatment. **P < *0.05 (Compared to untreated Anglne cell group); #*P *< 0.05 (compared with the HA-treated group).

Supplementary Figure 3 The mRNA expression of HRH1 **(A)** and HRH3 **(B)** after HA, AZD9496 DNP and APG treatment. **P < *0.05 (Compared to untreated OVCAR-3 cell group).

## Abbreviations

ERα, Estrogen receptor α; ERβ, Estrogen receptor β; MCs, Mast cells; APG, Apigenin; HIF-1, Hypoxia Inducible Factor -1; VEGF, Vascular Endothelial Growth Factor; RIN, RNA Integrity Number; PCA, Principal Component Analysis; VAMP-8, Vesicle associated membrane protein-8; NSF, N-ethyl maleimide sensitive factor; SNARE, Soluble N-ethyl maleimide sensitive factor adhesion protein receptor; LIF, Leukemia Inhibitory Factor; HDC, L-histidine decarboxylase; HRH1, Histamine H1 receptor; HRH3, Histamine H3 receptor.
