# Supplementary figures and images for: Apigenin Inhibits the Histamine-Induced Proliferation of Ovarian Cancer Cells by Downregulating ERα/ERβ Expression
Source: Front Oncol. 2021 Sep 8;11:682917. doi: 10.3389/fonc.2021.682917 (PMC8456091; doi:10.3389/fonc.2021.682917)

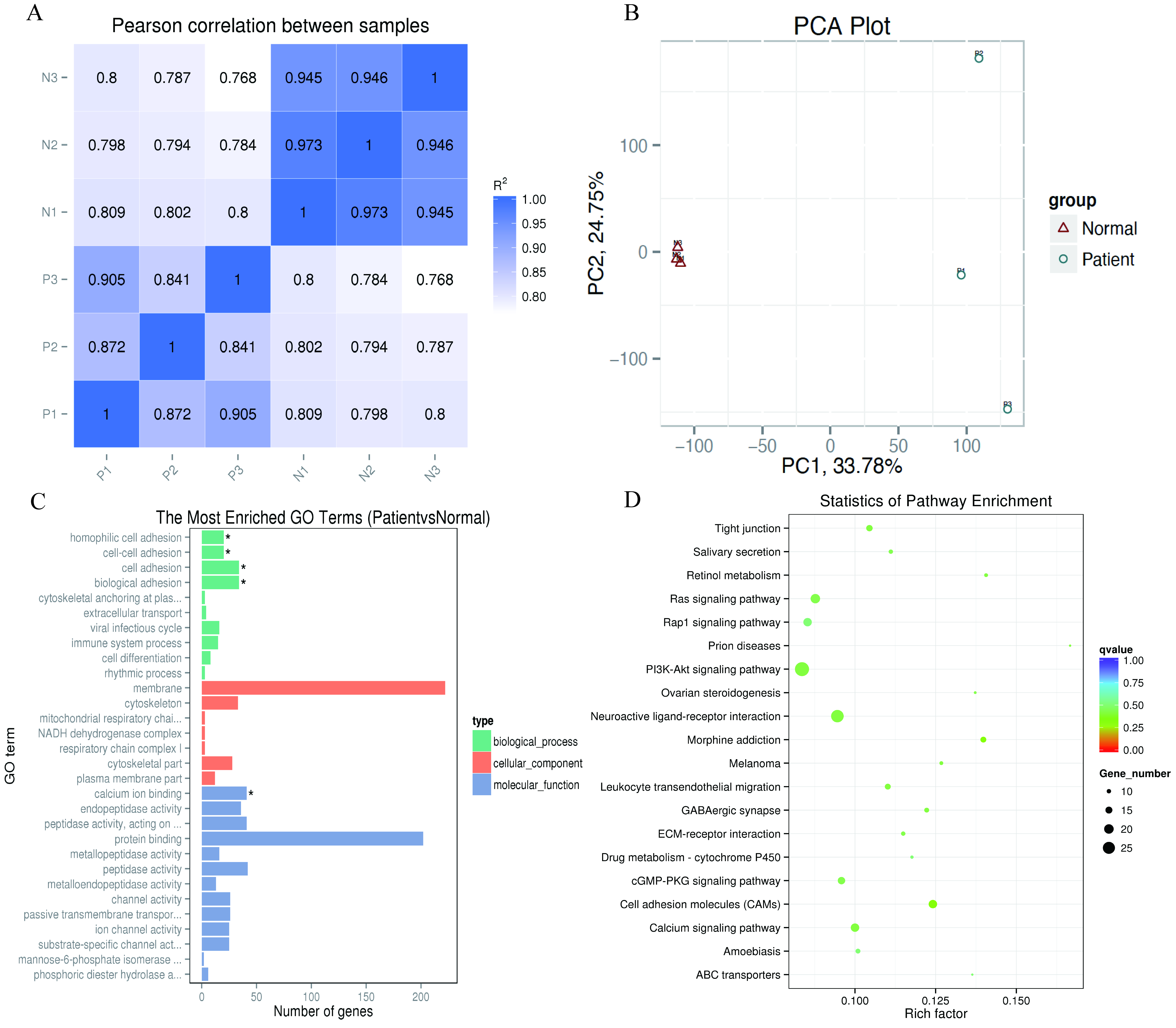

Supplement: Supplementary Figure 1 — Determination of DEGs between normal ovarian and ovarian cancer tissues though RNA-Seq analysis. (A) Correlation coefficient diagram between normal ovarian (n = 3) and ovarian cancer tissue samples (n = 3). (B) Principal Component Analysis diagram between ovarian tissue samples; (C, D) GO (C) and KEGG (D) enrichment analysis of DEGs. [file Image_1.tif]

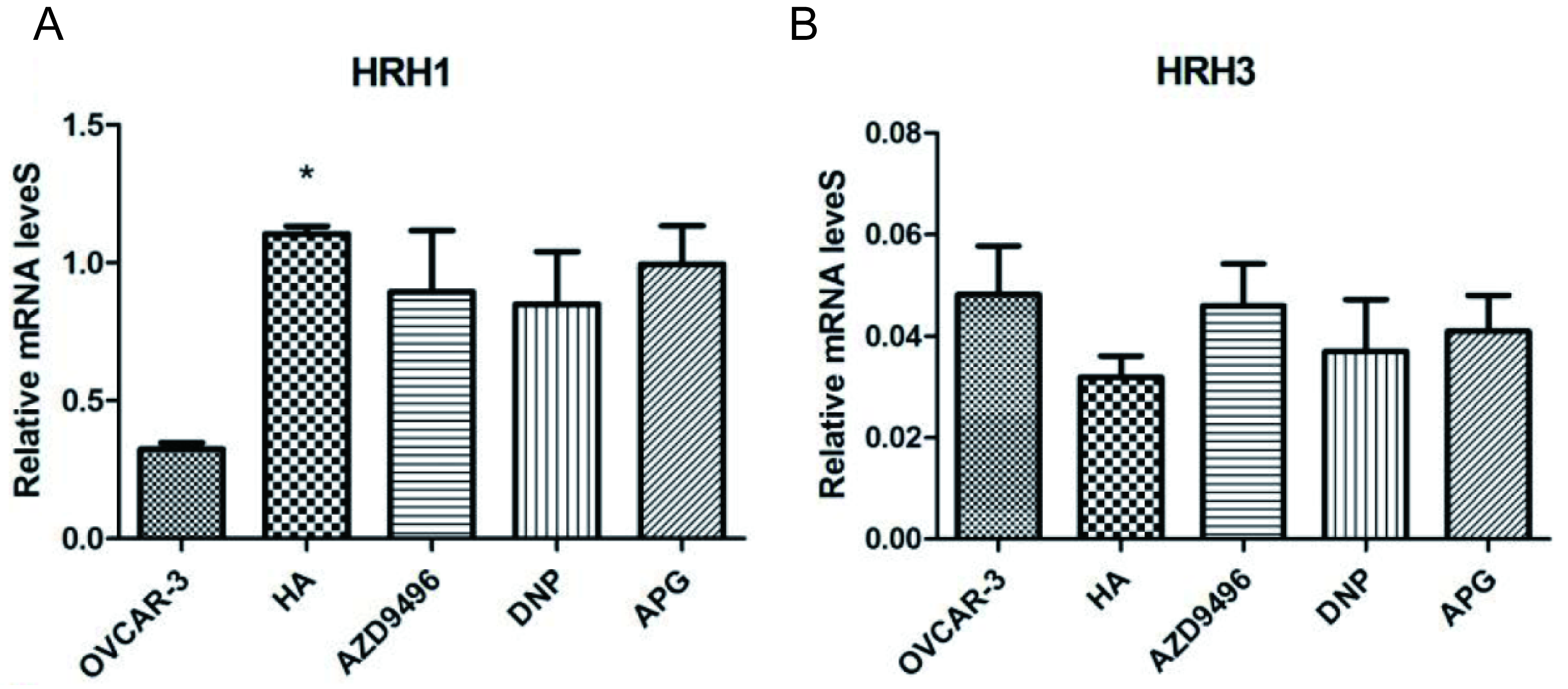

Supplement: Supplementary Figure 2 — The proliferation rate of Anglne cell after HA and APG treatment. *P < 0.05 (Compared to untreated Anglne cell group); #P < 0.05 (compared with the HA-treated group). [file Image_3.tif]

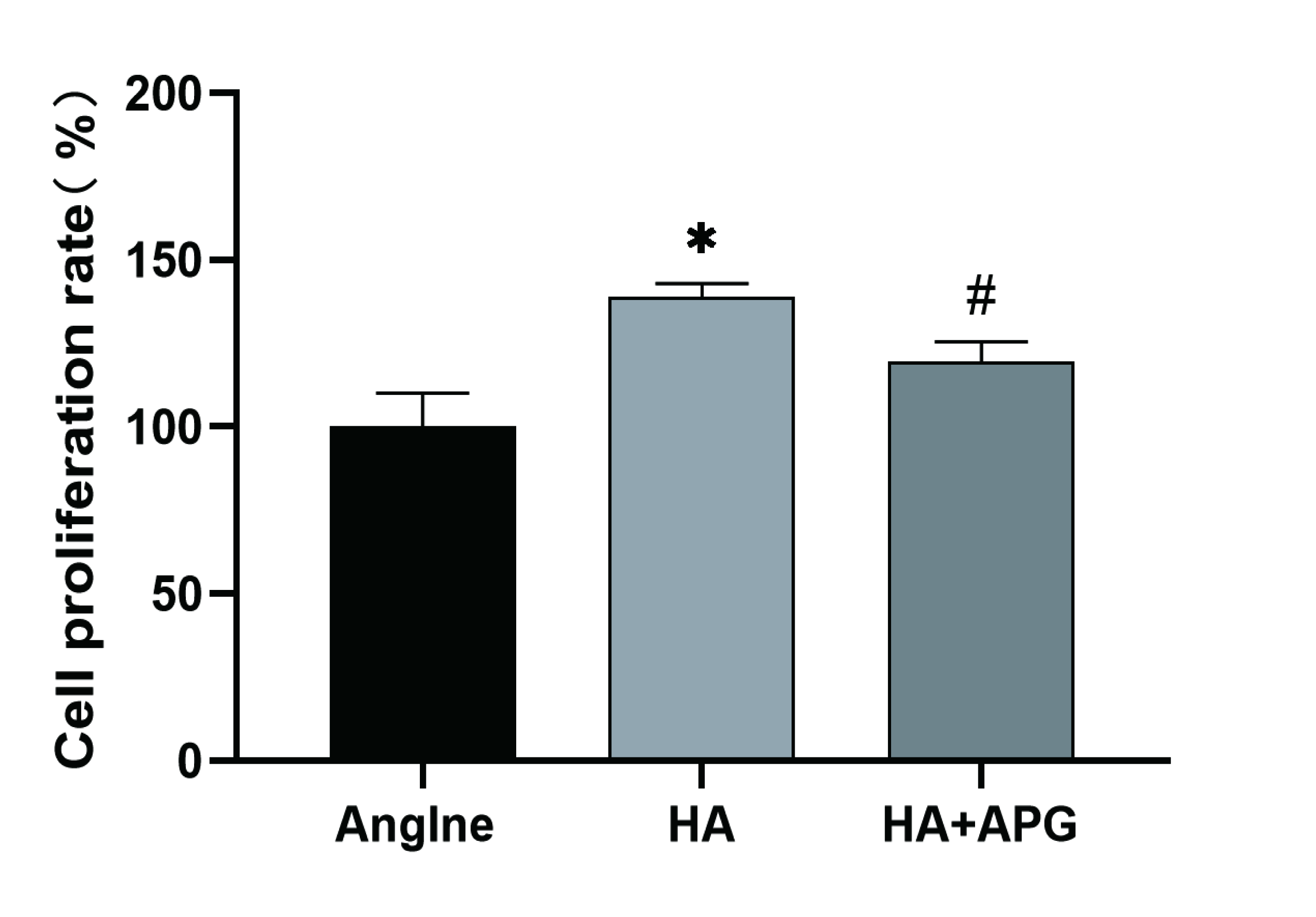

Supplement: Supplementary Figure 3 — The mRNA expression of HRH1 (A) and HRH3 (B) after HA, AZD9496 DNP and APG treatment. *P < 0.05 (Compared to untreated OVCAR-3 cell group). [file Image_2.tif]
