# Supplementary material for: Apigenin Inhibits the Histamine-Induced Proliferation of Ovarian Cancer Cells by Downregulating ERα/ERβ Expression
Source: Front Oncol. 2021 Sep 8;11:682917. doi: 10.3389/fonc.2021.682917 (PMC8456091; doi:10.3389/fonc.2021.682917)
Supplement: Supplementary file 4 [file DataSheet_1.docx]

**Supplementary Table 1 Donor Information**

|  | Ovarian Cancer Donor | Normal Ovarian Donor |
| --- | --- | --- |
| Number | 3 | 3 |
| Age | 30-50 | 30-50 |
| Diagnosis | Seromucous carcinoma | Granulose cell tumor |
| familial cancer history | No | No |
| Alcohol and tobacco addiction | No | No |
| Inspection tissue | Tumor infiltration | No tumor infiltration |

**Supplementary Table 2 Primer Sequences for RT-PCR**

| Gene | Primer sequences（5'-3') | | | Product(bp) |
| --- | --- | --- | --- | --- |
|  | Forward | | Reverse |  |
| β-actin | CTCGCCTTTGCCGATCC | GGGGTACTTCAGGGTGAGGA | | 258 |
| HRH1 | TGGTCACAGTAGGGCTCAAC | CAAGGTGGGCAGGTAGAAGT | | 497 |
| HRH3 | GCCATCCTGAGTTGGGAGTA | TCGTACCAGTAGTCGGGGAC | | 677 |
| ER α | ATGCGCTGCGTCGCCTCTAAC | CGCAGGGCAGAAGGCTCAGA | | 78 |
| ER β | AGCGCGGAGGCTGCGAGAAAT | CCTGCTCTTCGCCCTGCAAGTT | | 56 |

| GROUP | SAMPLE | RAW  SEQUENCE | FILTERED  SEQUENCE | ERROR  RATE | Q20（%） | Q30（%） | | GC CONTENT（%） | |
| --- | --- | --- | --- | --- | --- | --- | --- | --- | --- |
| normal ovarian tissue | N1 | 66295130 | 64212992 | 0.02 | 96.83 | 92.02 | | | 48.47 |
|  | N2 | 70422554 | 69197814 | 0.02 | 96.9 | 92.15 | | | 48.34 |
|  | N3 | 49154458 | 48109252 | 0.02 | 96.22 | 90.71 | | | 48.73 |
| ovarian cancer tissue | P1 | 62380436 | 61254148 | 0.02 | 96.78 | | 91.88 | | 49.69 |
|  | P2 | 85249398 | 83699958 | 0.02 | 96.84 | | 92.02 | | 49.51 |
|  | P3 | 59282110 | 57346706 | 0.02 | 96.72 | | 91.89 | | 48.44 |

**Supplementary Table 3 Quality Evaluation of RNA Sequencing Data**

| GENETIC ID | Log2FoldChange | p-VALUE | GENE SYMBLE |
| --- | --- | --- | --- |
| ENSG00000165215 | 8.555897738 | 2.04E-277 | CLDN3 |
| ENSG00000101443 | 8.219519349 | 2.40E-200 | WFDC2 |
| ENSG00000110195 | 7.937879854 | 1.82E-176 | FOLR1 |
| ENSG00000124107 | 7.919073998 | 3.55E-163 | SLPI |
| ENSG00000272398 | 7.705848251 | 3.81E-201 | CD24 |
| ENSG00000102854 | 7.412961273 | 8.91E-178 | MSLN |
| ENSG00000160678 | 7.33052172 | 8.07E-135 | S100A1 |
| ENSG00000189143 | 7.162009073 | 5.11E-247 | CLDN4 |
| ENSG00000128342 | 7.137790791 | 7.65E-204 | KRT7 |
| ENSG00000135480 | 7.048108142 | 6.54E-117 | LCN2 |
| ENSG00000147465 | -7.984533343 | 1.67E-203 | STAR |
| ENSG00000214548 | -7.609586398 | 2.64E-182 | MEG3 |
| ENSG00000112936 | -6.958796245 | 1.92E-120 | C7 |
| ENSG00000143125 | -6.786241205 | 2.01E-238 | PROK1 |
| ENSG00000258498 | -6.612217838 | 3.17E-271 | DIO3OS |
| ENSG00000173714 | -6.396031013 | 4.23E-260 | WFIKKN2 |
| ENSG00000185559 | -6.203123059 | 1.36E-95 | DLK1 |
| ENSG00000134201 | -6.073099727 | 5.32E-247 | GSTM5 |
| ENSG00000064205 | -5.8989659 | 1.19E-218 | CCN5 |
| ENSG00000188257 | -5.873784283 | 7.98E-103 | PLA2G2A |

**Supplementary Table 4 Top 20 Differentially Expressed Intersection Genes**
